# Supplementary material for: Patterns of Intron Gain and Loss in Fungi
Source: PLoS Biol. 2004 Nov 30;2(12):e422. doi: 10.1371/journal.pbio.0020422 (PMC532390; doi:10.1371/journal.pbio.0020422)
Supplement: Table S1 — Also available at http://genes.mit.edu/NielsenEtAl/. (4.3 MB ZIP). [file pbio.0020422.st001.zip › NielsenEtAl/html/2.html]

AN4942.1.NCU04759.1.MG02479.1.FG08477.1


```
 CLUSTAL W (1.82) Multiple Sequence Alignments - Introns Inserted


Sequence 1: AN4942.1	125 aa
Sequence 2: FG08477.1	125 aa
Sequence 3: NCU04759.1	119 aa
Sequence 4: MG02479.1	126 aa
Alignment Length: 130 aa
Number Identitical Residues: 40 aa
Alignment Score (without introns) 2353


MG02479.1 	MT-T1PQAVAT1EFVQFYYSEFDKGREARAAWSNLVY0TDQSVLTFESTEHRGKTAIAEK
NCU04759.1	MSLD1FTAIAT1QFVAHYYSTFDSDRKNLAG----LY0RDNSMLTFEGAQSLGAQGITEK
FG08477.1 	MAGN1FEEVAK1QFVEYYYNTFDSDRKGLNA----LY0RPNSMLTFESASVLGAEAIAEK
AN4942.1  	MA-D1FQSIAQ1QFVTFYYQTFDGNRAGLAP----LY0RDHSMLTFETSAIQGVAGIIEK
          	*:      :*  :** .**. ** .*         :*   :*:**** :   *  .* **

MG02479.1 	LS0GLPFEVVKHQVSTLDVQTT-VHDGIIILVTGQLL0VDEEQRPMNFSQVFQLLK-ADD
NCU04759.1	LT0-----KVKHEYGPPDAQPT-ATGGIIILVTGQLI0VDDEQRPLGYSQAFQLSQDASG
FG08477.1 	LV0SLPFQQVKHKVATLDAQPSNDQGGVIILITGALL0IDEEQNPMNFSQTFQLQRDQAG
AN4942.1  	LT0SLPFQKVQHQVSTLDAQPSGEHGGILVLVTGALL0VDEEKNPMNYTQTFQLMPDGAG
          	*  . . . *:*: .. *.*.:.  .*:::*:** *: :*:*:.*:.::*.***  .  .

MG02479.1 	RWYALNDVFKLVLG-
NCU04759.1	QWFVFNDIFKLVLL-
FG08477.1 	NYYVYNDLFKLVFG-
AN4942.1  	SYFVLNDVFRLIYNA
          	 ::. **:*:*:  :
```
